# Extensive hepatic portal venous gas and gastric pneumatosis in a cat

**DOI:** 10.1002/vms3.399

**Published:** 2020-11-22

**Authors:** Karin T. Spiller, Beth W. Eisenberg

**Affiliations:** ^1^ Yonkers NY USA; ^2^ Massachusetts Veterinary Referral Hospital Woburn MA USA

**Keywords:** computed tomography, feline, hepatic portal venous gas, pneumatosis

## Abstract

A 15‐year‐old female neutered Domestic Long Hair cat was presented for acute hematemesis. Initial diagnostic workup, including serum biochemistry panel, complete blood count and coagulation profile, was unremarkable. Abdominal ultrasound showed gastric mural thickening and non‐obstructive gastric foreign material. Endoscopy was performed to remove the foreign matter and obtain biopsies. Significant abnormalities of the upper gastrointestinal (GI) tract were not noted endoscopically. Overnight, the patient required a packed red blood cell transfusion following two episodes of severe hematemesis, hypotension and collapse. Serial radiographs and ultrasound revealed hepatic portal venous gas (HPVG). Computed tomography (CT) scan confirmed massive gas accumulation within the liver and emphysematous gastritis. The patient became increasingly unstable and, given her rapid decline, humane euthanasia was elected. Gastric and duodenal histopathology showed inflammatory changes, spirochetosis and mucosal epithelial degeneration. HPVG is a rarely described finding and prognosis varies drastically depending on aetiology. To the best of our knowledge, this is the first description of portal vein gas documented on multiple imaging modalities, including CT, in a cat. The patient in this report had several potential risk factors including prior endoscopy, compromise of the intestinal barrier and evidence of gastric mural bacterial invasion.

## Graphical abstract

This report describes a case of hepatic portal venous gas (HPVG) in a feline visualized initially on ultrasound and ultimately confirmed with CT, which is considered the gold‐standard for identification in humans. HPVG is associated with a variety of underlying disease processes, some of which require emergent aggressive intervention and may result in significant mortality. Reports of HPVG in veterinary medicine are sparse.

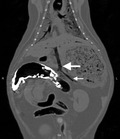

**Abbreviations**
CT: computed tomography
GI: gastrointestinal
HPVG: hepatic portal venous gas
IV: intravenous
PCV: packed cell volume
RI: reference interval
TP: total protein

## 1 INTRODUCTION

HPVG is an infrequently observed imaging finding that is associated with a number of conditions. It represents the accumulation of air within the portal vein and can be identified with a variety of imaging modalities. HPVG is not a diagnosis by itself, but rather a sign of an underlying disease process. In humans, it is seen with primarily with GI disease (intestinal or gastric dilatation secondary to ileus or obstruction, ulcerative disease or inflammatory conditions) or following procedures such as GI endoscopy. While non‐GI causes in people are less frequently encountered, HPVG has been found with colchicine toxicity (Saksena et al., 2003), seizures, and chronic obstructive pulmonary disease (Sebastia et al., 2000). HPVG has been described in veterinary patients with GI disease and following hydrogen peroxide administration (Faverzani et al., 2009). The basic mechanisms behind the development of HPVG are distension of viscera, compromise of either the gastric or intestinal walls and infectious or inflammatory states. The prognosis for patients with HPVG varies depending on the underlying aetiology. Here we document the rapid progression of HPVG and clinical deterioration in a feline using a combination of radiographs, ultrasound and CT.

## 2 CASE DESCRIPTION

A 15‐year‐old, neutered female Domestic Long Hair feline was presented to the Emergency Department with a 4‐day history of hyporexia and one episode of hematemesis. Physical exam was unremarkable and initial vitals were within normal limits. Complete blood count (Table 1) revealed a mild anaemia (haematocrit 28%; reference interval (RI) 30%–52%) and a normal platelet count. Prothrombin time and activated partial thromboplastin time (Table 2) were within reference ranges. Serum biochemistry panel (Table 3) showed a mildly elevated blood urea nitrogen (15 mmol/L; RI 5.7–12.9 mmol/L), normal creatinine and liver enzymes and a mild hypoalbuminemia (19 g/L; RI 23–39 g/L). Urine specific gravity was 1.047.

**TABLE 1:** Hematology

Parameter	Result, conventional units	Reference interval, conventional units	Result, SI units	Reference interval, SI units
White blood cells	15.3	4.2–17.0 × 10^3/µl	15.3	4.2–17.0 × 10^9/L
Platelets	163	155–641 × 10^3/µl	163	155–641 × 10^9/L
Neutrophils	11.6	2.3–13.6 × 10^3/µl	11.6	2.3–13.6 × 10^9/L
Lymphocytes	2.6	8.1–5.5 × 10^3/µl	2.6	8.1–5.5 × 10^9/L
Monocytes	1.0	0–0.6 × 10^3/µl	1.0	0–0.6 × 10^9/L
Eosinophils	0	0–1.9 × 10^3/µl	0	0–1.9 × 10^9/L
Red blood cells	6.0	6.5–12.2 × 10^6/µl	6.0	6.5–12.2 × 10^12/L
Hematocrit	28.9	30.3%–52.3%	28.9	30.3%–52.3%
Hemoglobin	8.2	9.8–16.2 g/dl	5.1	6.1–10.1 mmol/L
Mean cell volume	47.7	35.9–53.1 fl	47.7	35.9–53.1 fl
Mean corpuscular hemoglobin	13.5	11.8–17.3 pg	13.5	11.8–17.3 pg
Mean corpuscular hemoglobin concentration	28.4	28.1–35.8 g/dl	284	281–358 × 10 g/L
Red cell distribution width	20.8	15%–27%	20.8	15%–27%
Reticulocytes	22.4	3.0–50.0 × 10^3/µl	22.4	3.0–50.0 × 10^9/L
% Reticulocytes	0.4		0.4	

**TABLE 2:** Coagulation times

Parameter	Result	Reference interval
Prothrombin time	14	15–22 s
Partial thromboplastin time	98	65–119 s

**TABLE 3:** Biochemistry profile

Parameter	Result, conventional units	Reference interval, conventional units	Result, SI units	Reference interval, SI units
Glucose	131	71–159 mg/dl	7.2	3.9–8.8 mmol/L
Creatinine	1.0	0.8–2.4 mg/dl	88.4	70.7–212.1 µmol/L
Blood urea nitrogen	42	16–36 mg/dl	15	5.7–12.9 mmol/L
Phosphorus	4.4	3.1–7.5 mg/dl	1.4	1.0–2.4 mmol/L
Total calcium	7.6	7.8–11.3 mg/dl	1.9	2.0–2.8 mmol/L
Total protein	6.0	5.7–8.9 g/dl	60	57–89 g/L
Albumin	1.9	2.3–3.9 g/dl	19	23–39 g/L
Globulin	4.1	2.8–5.1 g/dl	41	28–51 g/L
Alanine aminotransferase	24	12–130 U/L	24	12–130 U/L
Alkaline phosphatase	<10	14–111 U/L	<10	14–111 U/L
Gamma glutamyl transferase	2	0–4 U/L	2	0–4 U/L
Total bilirubin	<0.1	0.0–0.9 mg/dl	<1.7	0–15.4 µmol/L
Cholesterol	50	65–225 mg/dl	1.3	1.7–5.8 mmol/L
Amylase	845	500–1500 U/L	845	500–1500 U/L
Lipase	323	100–1400 U/L	323	100–1400 U/L

Supportive care was instituted with intravenous Lactated Ringer's solution at 2.5 ml kg^−1^ hr^−1^ (Braun Medical), pantoprazole sodium 1 mg/kg IV q24h (Wyeth Pharmaceuticals), and ondansetron 0.2 mg/kg IV q12h (Accord Healthcare). Abdominal ultrasound performed by a board‐certified radiologist revealed a normal liver and intestinal tract. Abnormalities were limited to the stomach and included mild gastric mural thickening with the impression of hyperechoic foci in the mucosa, potentially representing pinpoint gastric ulceration. A short, linear, partially shadowing object was also noted within the gastric lumen; this non‐obstructive foreign matter was thought to be either hair or plant material. The patient's packed cell volume (PCV) had dropped to 19% and upper GI endoscopy was pursued following ultrasound to further evaluate possible GI haemorrhage as a source for her blood loss.

Endoscopy was performed by a board‐certified internist. The patient was pre‐medicated with butorphanol tartrate 0.3 mg/kg IV (Zoetis, Kalamazoo, MI), induced with Propofol 3.4 mg/kg IV (Zoetis) and maintained with Isoflurane (Zoetis). At induction for anaesthesia, a piece of tape was discovered adhered to the hard palate which was easily removed. The stomach contained several pieces of plastic which were also removed. Mild erosions were seen in the gastric mucosa and the duodenum had a mild cobblestone appearance; no ulcerations or other abnormalities were noted. Biopsies were obtained from the duodenum and stomach and submitted for histopathology. Recovery from the procedure was uneventful.

To definitively rule out a metallic foreign body not visualized on ultrasound or endoscopy, abdominal radiographs were performed on recovery. X‐rays showed a moderate volume of gas throughout the GI tract, consistent with prior endoscopy. PCV and total protein (TP) had fallen to 14% and 48 g/L and a single unit of type and crossmatch‐compatible packed red blood cells (6.8 ml/kg) was administered over 3.5 hr. PCV and TP 2 hr post‐transfusion were 19% and 50 g/L. A single 5 ml dose of barium sulphate suspension (E‐Z PAQUE, Bracco Diagnostics) was administered orally as additional gastroprotectant therapy.

**FIGURE 1:**
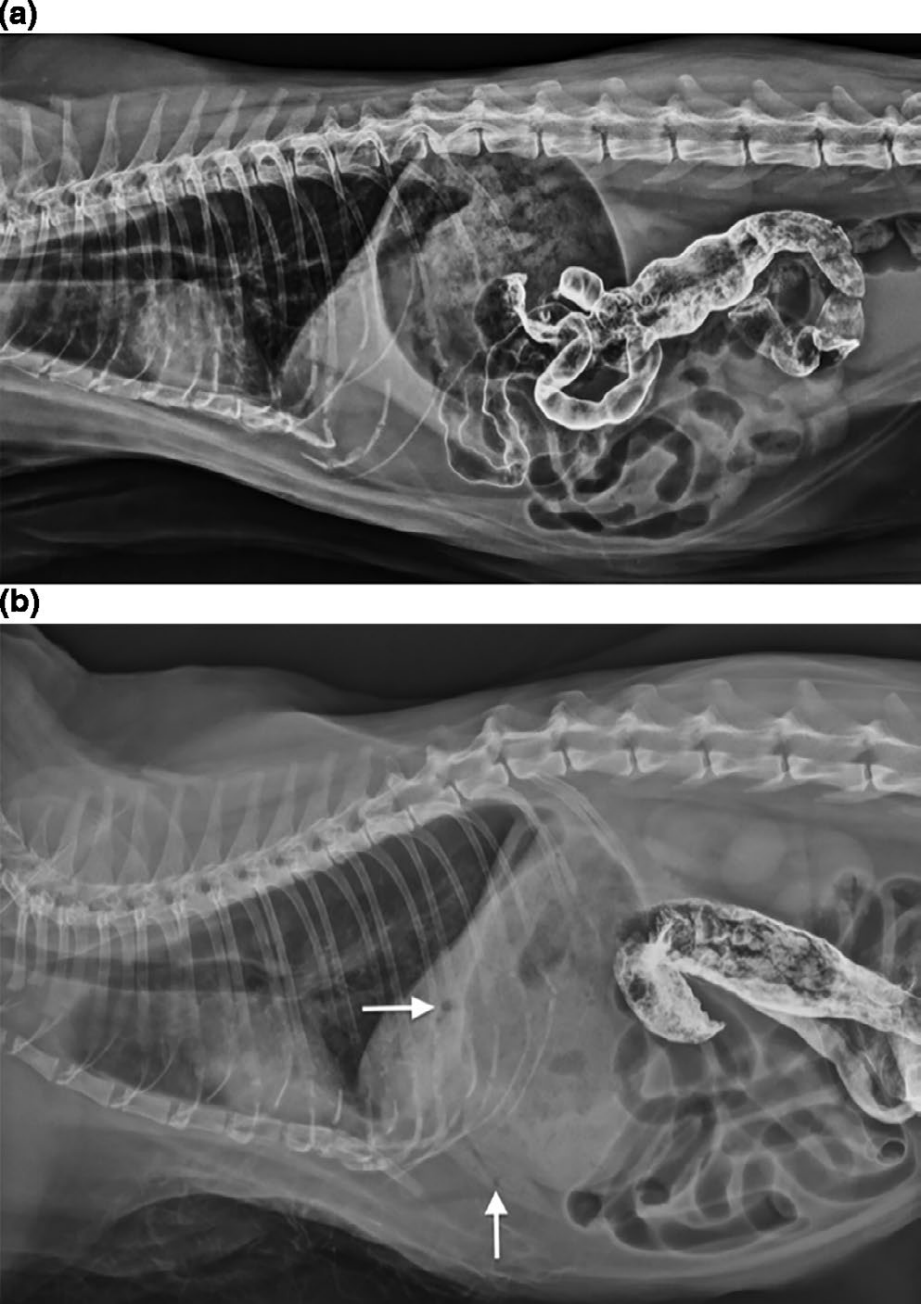
(a) Right lateral whole body radiograph. There is a moderate volume of gas in the esophagus, stomach, and intestinal tract. The gastric wall appears normal and smooth. No hepatic gas is visible. Barium is present in the jejunum, ileum, and colon. There is a bronchointerstitial pattern with faint patchy opacities that may represent recumbent atelectasis. (b) Right lateral whole body radiograph. Compared with radiographs performed 3 hr earlier, a reticular gas pattern has developed within the liver (arrows) and there is persistent gaseous distension of the GI tract. Barium is present in the distal small intestine and colon

The next morning the patient vomited a large volume of frank blood with clots. She became tachypneic (respiratory rate of 56 breaths/min) and was laterally recumbent and hypotensive (systolic blood pressure of 60 mmHg). Distension of the cranial abdomen was noted and abdominal radiographs revealed progressive gaseous distension of the stomach and intestines, concerning for severe ileus. The gastric wall appeared normal and barium was noted in the distal jejunum, ileum and colon (Figure 1a). A single crystalloid bolus of 20 ml/kg and an injection of 1 mg/kg IV maropitant citrate (Zoetis, Kalamazoo, MI) were administered. The patient's blood pressure improved to 105 mmHg, she appeared brighter and her tachypnoea resolved (respiratory rate of 24 breaths/ min).

Within a few hours, the patient experienced another episode of hematemesis, hypotension (blood pressure of 25 mmHg), tachypnoea (respiratory rate of 60 breaths/ min) and lateral recumbency. Antibiotic therapy was initiated with ampicillin sulbactam 30 mg/kg IV q8h, (Mitim S.R.L., Brescia, Italy), enrofloxacin 5 mg/kg IV q24h (Bayer, Shawnee Mission, KS) and metronidazole 10 mg/kg IV q12h (Hospira) for potential sepsis secondary to bacterial GI translocation. Repeat abdominal radiographs revealed hepatoportal venous gas as well as persistent gaseous distension of the stomach and intestines (Figure 1b).

**FIGURE 2:**
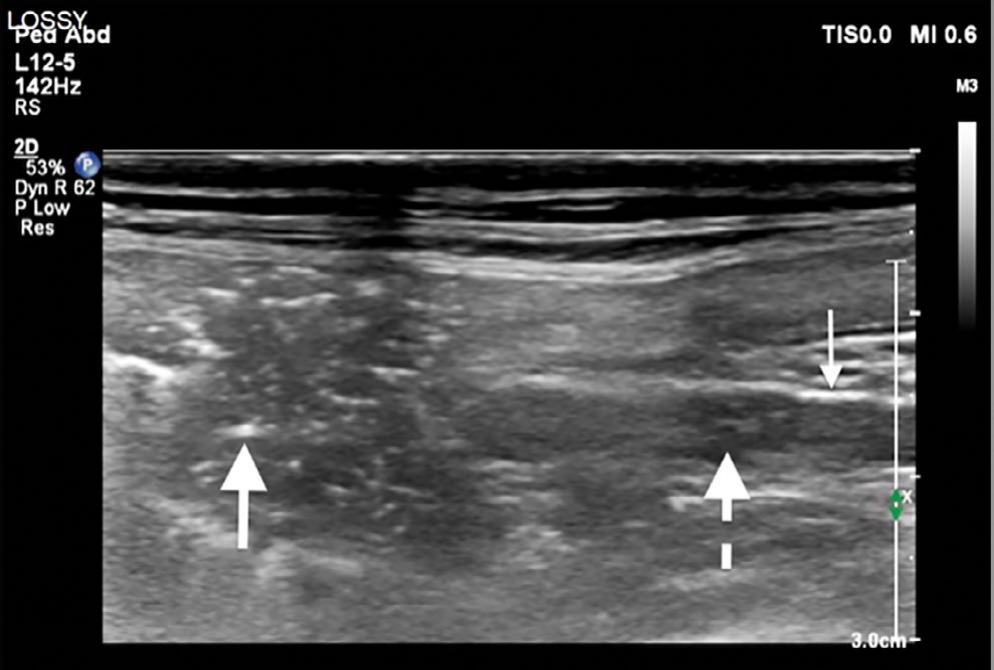
Ultrasound of the pre‐hepatic portal vein (right side of image) entering the liver. Hepatic emphysema is seen as hyperechoic foci within the hepatic parenchyma (large arrow) and associated reverberation artifact (not well‐visualized in the image). Gas bubbles within the lumen of the portal vein are seen as strongly echogenic particles (dashed arrow). Gas shadowing (small arrow) with associated comettail artifact represents bubbles within the non‐dependent portion of the portal vein

**FIGURE 3:**
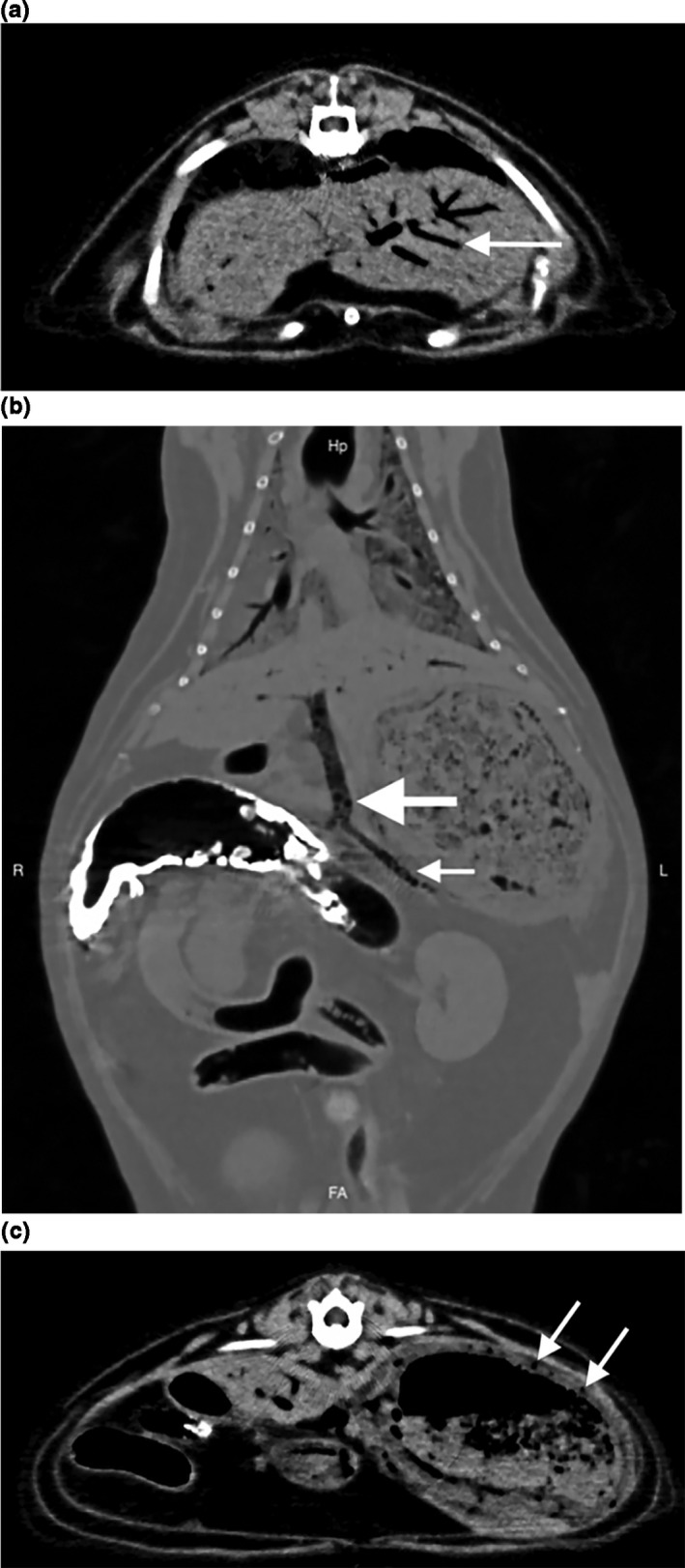
(a) Whole body CT showing marked gas accumulation in multiple locations. Transverse image. There is an extensive dendritic hypoattenuating pattern throughout the liver (arrow), consistent with intravascular gas. (b) Whole body CT showing marked gas accumulation in multiple locations. Dorsal image. Multiple gas bubbles are visible within the lumen of the splenic vein (small arrow) as it enters the portal vein (large arrow). Residual barium remains in the transverse colon. (c) Whole body CT showing marked gas accumulation in multiple locations. Transverse image. There is mild to moderate thickening of the gastric wall along with thin linear chains of intramural gas bubbles (arrows)

An abdominal ultrasound confirmed gas within the liver and portal vein but the origin could not be identified (Figure 2). A large amount of gas was visible in the parenchyma of more than one hepatic lobe with an extensive dendritic pattern. The gas could not be confirmed to be within biliary canaliculi or peripheral venous structures but clearly contrasted with the anechoic fluid within the gallbladder. A marked amount of non‐dependent gas bubbles were observed coursing in a cranial direction within the splenic and pre‐hepatic portal veins. Evaluation of the gastric wall was limited by the hepatic gas shadowing but subtle changes were noted, including less distinct mural layering and a more hypoechoic appearance compared with the previous exam. The patient again became markedly hypotensive (blood pressure of 40 mmHg) and unresponsive; intensive care with the addition of vasopressors and plasma and red cell transfusions was offered. Due to the patient's severe and rapid decline, the owner elected humane euthanasia and declined post‐mortem evaluation. CT scan performed without sedation immediately preceding euthanasia revealed extensive HPVG and a dendritic hypoattenuating pattern throughout the liver consistent with intravascular gas (Figure 3). A large amount of gas was present in all tributaries of the portal vein in addition to the colic veins, gastrosplenic veins and caudal thoracic vertebral veins. Mild to moderate thickening of the stomach wall was noted with thin linear chains of intramural gas bubbles radiating outward, most pronounced along the lesser curvature.

Gastric histopathology showed mild lymphoplasmacytic neutrophilic gastritis, minimal mucosal compromise, mild mucosal fibrosis and glandular nesting. Mild spirochetosis was seen in association with lymphocytic inflammation of the gastric pits. Duodenal changes included moderate neutrophilic lymphoplasmacytic enteritis and mucosal fibrosis. The heavy presence of neutrophils and surface epithelial degeneration suggested compromise of the mucosal barrier and a bacterial component. Although a definitive cause for the clinical deterioration was not found, the HPVG was suspected to be gastric in origin given that the majority of pathology was seen in the stomach.

## 3 DISCUSSION

Hepatic portal venous gas was originally reported in 1955 as a radiographic finding in infants with necrotizing enterocolitis (Wolfe & Evans, 1955). It refers to gas within the portal vein and is an infrequently documented type of pneumatosis. Pneumatosis, or the abnormal accumulation of air in the body, can also be found within the walls of the GI tract including the stomach or small and large intestines. Gastric pneumatosis can be further categorized as either emphysematous gastritis (EG) or gastric emphysema (GE) based on several factors (Parikh et al., 2015). While GE and EG produce similar radiographic changes, a combination of clinical signs, systemic abnormalities and endoscopic appearance of the stomach wall can be used to differentiate between the two clinical syndromes. A recent veterinary case series found that patients with EG had severe clinical signs and significant clinicopathologic abnormalities such as anaemia or hypoalbuminemia (Thierry et al., 2019). Emphysematous gastritis was associated with a poor outcome in both veterinary and human studies (Parikh et al., 2015; Thierry et al., 2019). The clinical picture of the feline in this report is most consistent with EG, as her signs were severe and lead to a poor outcome.

Portal vein gas can be visualized with a variety of imaging modalities, including radiographs, ultrasound and computed tomography. On plain radiographs, HPVG is described as branching radiolucencies directed peripherally towards the liver capsule from the region of the main portal vein. This pattern is due to centrifugal blood flow carrying gas to the periphery (Allaparthi & Anand, 2013). Given that large amounts of gas are needed to produce visible changes, radiography is an insensitive method for detecting HPVG. False negatives have been reported in up to 80% of cases (Kesarwani et al., 2009).

On ultrasound, descriptions of HPVG vary slightly depending on location and pattern of distribution. Within the portal venous system it is seen as gas bubbles coursing in the direction of blood flow (Naguib et al., 2012). In the non‐dependent portions of the hepatic parenchyma, HPVG manifests as either bubbles or gas shadowing (Lee et al., 1993). Differentiation between parenchymal and portal vein gas was recently described in a veterinary study of patients with hepatic emphysema; a broad term for gas found within the parenchyma, portal venous system or biliary tree. Both were visualized ultrasonographically as highly echogenic particles, with parenchymal gas producing reverberation artifacts within focal areas of the liver compared to PVG seen as mobile artifacts with a more peripheral location (Manfredi et al., 2019).

HPVG has been categorized using three patterns in an attempt to determine whether imaging characteristics could be used to predict patient outcome. The patterns were described as either dot‐like, streak‐like, or fruit‐pulp‐like, representing increasing amounts of gas within the portal system. The authors concluded that streak‐like and pulp‐like patterns are less likely to be associated with benign aetiology and, therefore, carry a worse prognosis compared to dot‐like patterns of HPVG (Pan et al., 2007). The extensive intravascular gas in the patient described here would have been most consistent with a fruit‐pulp‐like pattern and a high likelihood of non‐survival. CT is considered the gold‐standard in human medicine to identify HPVG as it allows visualization of smaller quantities of air compared to plain radiography. It may also provide more information about the primary aetiology, especially in cases of intestinal ischaemia (Wiesner et al., 2003).

The exact mechanism of portal venous gas accumulation is unknown. Predisposing factors can be divided into several broad categories, including damage to the GI mucosal barrier, intra‐abdominal infections, idiopathic and secondary to increased or acute pressure changes within the abdominal cavity or GI tract. Migration of gas across an intact mucosal barrier is possible if there is an increase in intraluminal pressure, seen secondary to GI distension. Pathologic causes of distension include ileus and mechanical obstruction from neoplasia or gastric‐dilatation and volvulus. Gas movement across intestinal or gastric walls into circulation is facilitated if the mucosal barrier is compromised. Mucosal injury can occur with ulcerative diseases, ischaemia, infection and inflammatory conditions such as inflammatory bowel disease. In humans, the most commonly cited cause of HPVG is intestinal ischaemia secondary to thromboembolism, hypoperfusion or bowel obstruction (Sebastia et al., 2000). HPVG has also been noted following certain procedures such as endoscopy, laparoscopy or barium enema administration. A small percentage of humans with blunt abdominal trauma are found to have ultrasonographic evidence of venous gas, thought to be the result of rapid increases in intra‐abdominal pressure forcing intraluminal air across intestinal walls and into circulation (Brown et al., 1999). Infections and inflammatory conditions, including intra‐abdominal abscesses, pancreatitis, cholecystitis and enterocolitis have been documented in patients with HPVG. Proposed mechanisms include intraluminal or submucosal bacterial gas production leading to migration into the bloodstream. HPVG is considered idiopathic in approximately 15% of cases (Sebastia et al., 2000).

Management of patients with HPVG is focused primarily on treating the underlying disease process. Historically, HPVG was most commonly associated with intestinal necrosis and carried high mortality rates of 75%–85%. Surgery was always recommended except in cases of ulcerative colitis (Abboud et al., 2009; Liebman et al., 1978). Given the broad range of associated conditions, it can be difficult to identify which cases require surgical intervention. Serial imaging studies to document progression may aid in determining if surgery is warranted. A recent study evaluated the benefits of serial CT scans to assess progression of HPVG (Higashi et al., 2015). Patients without signs of intestinal ischaemia or peritoneal irritation were categorized as not requiring emergency surgery and thus conservatively managed. These patients had a recheck CT performed every 5 hr, and the amount of gas decreased rapidly within a few hours. Veterinary specific limitations such as finances and multiple rounds of sedation may make this strategy impractical; however, it may help avoid an unnecessary surgery. Non‐surgical options should be considered if there is no evidence of ischaemia or in patients with ulcerative or inflammatory diseases without clinical deterioration. Medical management typically involves fluid therapy, broad‐spectrum antimicrobials, analgesics and gastroprotectants.

The prognosis for patients varies widely and is dependent on the underlying mechanism for the development of HPVG. Earliest published mortality rates in humans were high but most of these cases cited an ischaemic aetiology, which typically carries a poor prognosis. More recent studies document lower mortality rates ranging from 19%–29% (Faverzani et al., 2009; Huang et al., 2014). Several factors may be responsible for the apparent improvement in survival. Advances in imaging techniques and routine use of more sensitive modalities have enabled detection of smaller accumulations of gas. The majority of initial reports identified HPVG radiographically and, since large volumes of air must be present to be seen on plain films, these cases likely represent either very severe or advanced disease. The ability to identify subtle changes may allow more timely intervention and thus better outcomes.

HPVG descriptions in veterinary medicine are limited to a handful of case reports and a single retrospective study. Portal vein gas was documented in a dog secondary to hydrogen peroxide administration for the induction of emesis. HPVG was identified sonographically 18 hr after the attempted emesis, and ultrasound 1 week later showed resolution of the gas (Faverzani et al., 2009).

Another report described HPVG visualized sonographically in a young dog with severe ulcerative colitis. Medical management with antibiotics and antiemetics resulted in initial clinical improvement. Recheck ultrasound 1 week after presentation demonstrated resolution of the gas accumulation. Treatment was continued but the patient was ultimately euthanized 2 weeks later due to acute deterioration and possible sepsis secondary to a resistant bacterial infection (Cartwright et al., 2016).

A recent case report described a 9‐month‐old feline with emphysematous hepatitis and suspected HPVG (Hutchinson et al., 2018). Notable laboratory abnormalities included markedly prolonged clotting times and severe non‐regenerative anaemia. Abdominal radiographs revealed gas within the hepatic and splenic parenchyma, intestinal walls and suspected HPVG. Euthanasia was elected shortly after presentation due to the severity of clinical signs and perceived guarded prognosis. Necropsy performed approximately 18 hr after death revealed a diffusely abnormal liver and gas‐distended intestines with intraluminal haemorrhage. Histopathology showed hepatic periportal emphysema and bowel culture produced *Clostridium perfrigens* and *Escherichia coli*. The significance of the culture results is unknown as both are normal intestinal flora and there was a delay in sample collection.

Another case report described a feline with acute vomiting and a markedly elevated alanine transaminase. Abdominal radiographs showed gas within the intestinal walls and presumed portal venous gas. Abdominal ultrasound, however, did not find evidence of gas within the liver or associated vasculature. Necrotizing haemorrhagic enterocolitis secondary to *Clostridium difficile* and multifocal acute hepatocellular necrosis with haemorrhage were found at necropsy (Walczak et al., 2018). *Clostridium* is a frequently noted pathogen in humans with emphysematous gastritis (Parikh et al., 2015), but the clinical significance of this finding in veterinary patients is uncertain. The cat presented in our report did not have a necropsy or faecal examination for bacterial toxins performed, so it is possible a clostridial component was missed.

The origin of the HPVG in this patient was not definitively identified but was most likely due to the gastric pathology. The possibility of an intestinal component, such as inflammatory bowel disease or GI lymphoma, cannot be excluded. However, primary consideration is given to a gastropathy since the majority of the abnormalities were seen in the stomach. In a recent retrospective, all patients with HPVG were noted to have some type of abdominal pathology (GI disease, peritonitis, intra‐abdominal abscess) (Manfredi et al., 2019). Distension of the GI tract secondary to ileus and endoscopy may have forced air across the walls of the stomach and into circulation. Compromise of the mucosal barrier due to underlying inflammatory disease and gastric foreign material potentially facilitated gas migration. Another possible mechanism for the development of HPVG is bacteraemia, for example from the transfusion of a contaminated red blood cell unit. The patient was noted to decline shortly after receiving the transfusion, but this association may only be temporal. Additional blood transfusions for her progressive anaemia and more aggressive fluid therapy may have helped stabilize the patient, but likely would not have corrected the underlying pathology. Bacterial culture from the patient's blood, transfused unit or IV catheter, were not performed to support bacteraemia as a cause of the HPVG. Direct inoculation of gas into the bloodstream from injection of medications or flushing of the IV catheter is an unlikely explanation for the portal vein gas. Venous air embolism can potentially lead to circulatory collapse and death, depending on the amount of gas and rate of accumulation. Although the volume was extensive, the gas itself is not thought to be responsible for the patient's decline, as CT did not demonstrate any air within the heart or pulmonary vessels. While frequently associated with high mortality and rapid progression in humans, an ischaemic aetiology is considered unlikely, as there was no definitive evidence of ischaemia on CT or ultrasound in this patient. Since the HPVG was not found on initial ultrasound, a complication of the endoscopic exam cannot be completely ruled out as a cause for the patient's signs. Sepsis from an undocumented source remains possible, with the GI tract being the most probable nidus. Gastric spirochetosis was found on histopathology, but these bacteria are not gas‐producers nor are they typically highly pathogenic in cats. A notable limitation of this report is the absence of post‐mortem evaluation at necropsy that would have allowed for more extensive sampling of the liver and GI tract. The biopsies were obtained endoscopically and thus only partial‐thickness, so the presence of other aetiologies may have been missed.

In summary, treatment and prognosis for patients with HPVG are variable and largely dependent on the primary aetiology. Given the severity of changes needed to visualize HPVG on radiographs, CT may be the preferred imaging modality to detect subtle or early gas accumulation. CT scans performed in‐house can be reviewed by radiologists on site or sent out for interpretation, which improves access to specialists via telemedicine. Increasing availability in veterinary clinics and minimal time to obtain high‐quality diagnostic images are other benefits of CT that make it a valuable tool in the workup of cases with suspect HPVG. The patient in this report had several possible explanations for the reported HPVG including prior endoscopy, documented inflammatory changes and bacterial invasion of the gastrointestinal wall with compromise of the mucosal barrier. While none of these are typically associated with high mortality, the patient declined significantly and the primary cause for her signs remains unknown. HPVG is a rare finding in cats and the use of multiple imaging modalities to document its presence has not previously been reported.

## CONFLICT OF INTEREST

The authors report no conflict of interest.

## AUTHOR CONTRIBUTION

Karin Spiller: Writing‐original draft; Writing‐review & editing. Beth Eisenberg: Writing‐original draft; Writing‐review & editing.

### PEER REVIEW

The peer review history for this article is available at https://publons.com/publon/10.1002/vms3.399.
